# Integration of metal-organic frameworks into an electrochemical dielectric thin film for electronic applications

**DOI:** 10.1038/ncomms11830

**Published:** 2016-06-10

**Authors:** Wei-Jin Li, Juan Liu, Zhi-Hua Sun, Tian-Fu Liu, Jian Lü, Shui-Ying Gao, Chao He, Rong Cao, Jun-Hua Luo

**Affiliations:** 1 State Key Laboratory of Structural Chemistry, Fujian Institute of Research on the Structure of Mater, Chinese Academy of Science, Fuzhou 350002, P.R. China; 2 Collaborative Innovation Center of Chemistry for Energy Materials (2011-iChEM), Xiamen 361005, P.R. China; 3 University of Chinese Academy of Sciences, Beijing 100049, P.R. China

## Abstract

The integration of porous metal-organic frameworks onto the surface of materials, for use as functional devices, is currently emerging as a promising approach for gas sensing and flexible displays. However, research focused on potential applications in electronic devices is in its infancy. Here we present a facile strategy by which interpenetrated, crystalline metal-organic framework films are deposited onto conductive metal-plate anodes via *in situ* temperature-controlled electrochemical assembly. The nanostructure of the surface as well as the thickness and uniformity of the film are well controlled. More importantly, the resulting films exhibit enhanced dielectric properties compared to traditional inorganic or organic gate dielectrics. This study demonstrates the successful implementation of the rational design of metal-organic framework thin films on conductive supports with high-performance dielectric properties.

## Abstract (web summary)

The integration of porous metal-organic frameworks into devices for electronic applications is in its infancy. Here, the authors deposit metal-organic framework films onto conductive metal plate electrodes, and show that the resulting films exhibit enhanced dielectric properties.

## Graphical abstract

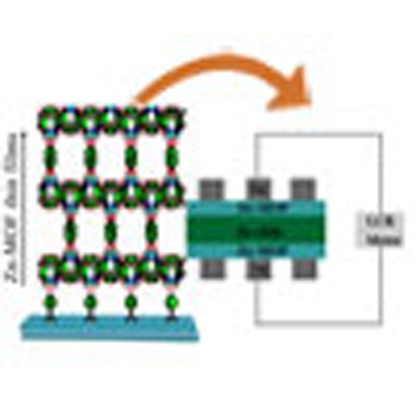

Considerable efforts have been devoted to preparing high-dielectric-constant (high-*κ*) materials with low leakage and a high breakdown strength as functional devices, such as thin-film transistors and high-electron-mobility transistors^1^. Conventional silicon-based electronics have been dominating as gate dielectric materials^2^,^3^; however, their relatively low dielectric constant and large static power dissipation limit their practical application. Therefore, exploitation of new high-*κ* materials that allow for a proportional increase in the gate thickness remains a challenge for the design of gate dielectric materials^4^,^5^. Inorganic hafnium dioxide (HfO_2_) and zirconium dioxide (ZrO_2_) have been widely studied as dielectric materials with high *κ* values. However, these materials are too brittle to be compatible with substrates, and the deposition techniques typically require high temperatures and expensive vacuum equipment^6^. Organic polymers have been deposited onto flexible plastic substrates to address this issue; however, the obtained organic polymer thin films typically suffer from low dielectric constants because of weak intermolecular forces in the gate devices. Moreover, organic polymer thin films typically have limited thermal management due to their modest thermal stability^7^,^8^,^9^.

Recently, organic and inorganic hybrid materials have emerged as a new type of electronic material that combines the distinctive properties of the high-κ of metal oxides and the flexibility of organic molecules^10^. However, one of the major challenges in the fabrication of these films is the low compatibility of the organic and inorganic components, which typically leads to the generation of defects in the resulting films and a significant decrease in device performance^11^.

As a hybrid material, metal-organic frameworks (MOFs) have garnered significant interest because their crystalline porous structures can be easily designed and functionalized through judicious choices or modifications of the metal nodes and organic linkers^12^,^13^,^14^,^15^,^16^,^17^. This approach provides an avenue for the rational design of a gate device on the nanoscale through electrochemical deposition of MOFs onto substrates. The coordination interactions of the metal centre and organic linkers endow the hybrid components with good compatibility and prevent the formation of defects on the self-assembled multilayers. In fact, good compatibility between the integrated MOF films and the surface of the substrate is imperative for their practical application in electronic devices^18^,^19^,^20^,^21^,^22^. To the best of our knowledge, although the ferroelectric properties of MOFs have been studied, investigations concerning control of the mechanical properties of porous MOFs, especially their dielectric constants, have been limited^23^,^24^. The dielectric constants of MOFs without guest molecules are typically small and independent of temperature because the framework does not have positional freedom in the crystalline state; however, polar guest molecules in such porous materials are typically highly free to move. Therefore, MOF materials with large dielectric constants could be achieved by entrapping or coordinating guest molecules with high polarizability^25^,^26^. However, preventing the loss of guest molecules is another issue that must be addressed. An interpenetrated MOF structure would enhance the binding of guest molecules with the framework^27^,^28^,^29^. Therefore, an ingenious combination of porous backbones and polar guest molecules could synergistically affect the polarizability of MOFs, resulting in materials that exhibit unique dielectric behaviours that they do not exhibit as a single component or amorphous polymer^18^,^30^. To the best of our knowledge, although MOFs, especially conductive MOFs, have attracted considerable attention because of their promising applications in electronic devices, the rational design of MOF structures on conductive supports to improve the electronic performance has not been previously reported.

Herein, a zinc MOF material based on a flexible ligand (i.e., 1, 3, 5-tris[4-(carboxyphenyl)oxamethyl]-2, 4, 6-trimethylbenzene (H_3_TBTC)), {[H_2_N(CH_3_)_2_][Zn(TBTC)]}·2DMF·EtOH (**1**), is designed and synthesized via a solvothermal reaction. **1** is a 2-fold interpenetrating anionic network with H_2_N(CH_3_)^2+^ counterions located inside the pore. The existence of the interpenetrating structure and the asymmetric cations that can induce dipole moments encourage us to investigate the dielectric properties of thin films of **1** (ref. 31). Inspired by Müller and co-workers' studies of the fabrication of bulk MOFs by electrochemical anodic oxidation of metal electrode^32^, Dinc and co-workers' results on the electrochemical reductive synthesis of MOF thin films^33^, De Vos as well as Ameloot and co-workers' studies of the fabrication of MOF thin films by electrochemical anodic oxidation of metal plates^34^, we deposit interpenetrated compound **1** onto a zinc plate using a temperature-controlled electrochemical anodic oxidation method. The proposed temperature-controlled electrochemical anodic oxidation method combines the merits of the electrochemical anodic oxidation method and the electrochemical reductive protonation of organic ligand method. Therefore, compound **1** is integrated onto a zinc plate via the fast production of Zn^2+^ from metal plates with simultaneous protonation of the ligand under mild conditions. After the deposition of **1** on the zinc plate using a facile electrochemical assembly approach, the morphology and thickness of the MOF thin film can be tuned by the temperature, reaction time and voltage. More importantly, the obtained thin film exhibits a dielectric constant that is more than three times higher than that of the bulk MOF material. In this study, we successfully demonstrate that the interpenetration of the structure effectively improved the dielectric properties of the MOF thin film. This phenomenon is further confirmed by experimental and theoretical studies of the dielectric properties of another pair of MOFs with a non-interpenetrated structure (MOF-123) and interpenetrated structure (MOF-246). In this study, we systematically investigate the relationship between the structure and the dielectric properties; the results may provide a new perspective for the design of electronic devices.

## Results

### Crystal structure of {Zn(TBTC) [H_2_N(CH_3_)_2_]}·2DMF·EtOH (1)

**Figure 1:**
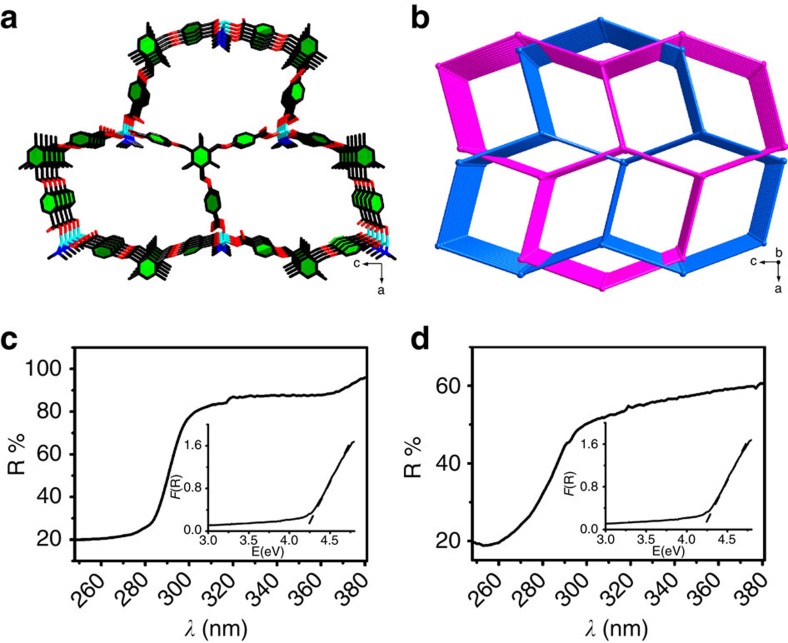
Crystal structure of 1 and experimental energy-band structures of 1 and thin film. (**a**) 3D framework of **1**. (**b**) Schematic of the 2-fold interpenetrated architectures of **1**. (**c**) UV–vis diffuse reflection spectrum of powder **1**; inset: UV–vis absorption spectrum of **1**. (**d**) UV–vis diffuse reflection spectrum of an as-prepared MOF thin film; inset: UV–vis absorption spectrum of an as-prepared MOF thin film.

Compound **1** crystallized in the *Pbcn* space group. The asymmetric unit of **1** contains a Zn^2+^ cation, a dimethylammonium (H_2_N(CH_3_)_2_^+^), a fully deprotonated TBTC^3−^ ligand, two uncoordinated *N*,*N*-dimethylformamide (DMF) molecules and one ethanol molecule (Supplementary Fig. 2, Supplementary Fig. 3, Supplementary Data 1). As shown in Supplementary Fig. 1, each Zn^2+^ is coordinated to three carboxyl O atoms from three different TBTC^3−^ and one N atom from a H_2_N(CH_3_)_2_^+^; therefore, TBTC^3−^ is considered to be a tri-bridging ligand that links the metal centres to the 3D network structure featuring 1D (13 × 17 Å) channels along the *b*-axis (Fig. 1a). The two aforementioned identical networks appeared in the same crystal lattice of compound **1**, which resulted in an overall 2-fold interpenetrated framework (Fig. 1b). The solvent-accessible volume of **1** was 4370.8 Å^3^ per unit cell, which is 44.4% of the total crystal volume calculated using the PLATON software^35^. The UV–vis diffuse reflection spectrum confirmed that the bulky MOF material exhibited an experimental energy gap of 4.24 eV (Fig. 1c). Materials with such a relatively large, broad energy gap (energy gaps of typical inorganic microelectronic materials: Si 1.1 eV, Pr_2_O_3_ 3.9 eV, HfO_2_ 5.6 eV) are believed to be good candidates for use as microelectronic materials^36^.

### Characterization of MOF thin films

**Figure 2:**
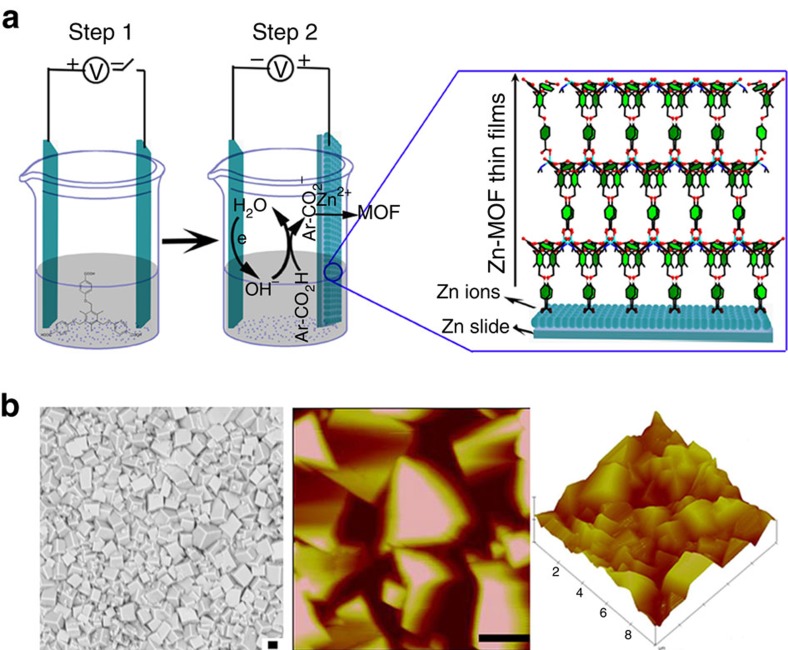
Representation of MOF film assembly concepts and morphology study. (**a**) Schematic of the electrochemical assembly of MOF thin films. (**b**) SEM image and typical height AFM image. Scale bar, 1 μm (**a**,**b**).

The MOF film was prepared using an electrochemical assembly method (Fig. 2a). Briefly, after electrolysis started, Zn^2+^ ions were produced *via* anodic oxidation on the surface of the Zn plate (anode). In addition, the TBTC^3−^ anions moved to the anode driven by the electric field. Then, the self-assembly of Zn^2+^ and TBTC^3−^ occurred, which resulted in deposition of a {[H_2_N(CH_3_)_2_] [Zn(TBTC)]}·2DMF·EtOH thin film onto the anodic Zn plate (Supplementary Fig. 4).

PXRD was carried out to evaluate the phase and crystallinity of the as-prepared MOF thin films (Supplementary Fig. 5). The peaks in the resulting diffractogram of the film match those for bulk MOF powder.

Attenuated total reflection infrared (ATR-IR) spectra were recorded to confirm the composition of the MOF thin films. The ATR-IR spectra of the as-prepared MOF thin films are consistent with that of the bulk material (Supplementary Fig. 6). The characteristic absorbance at 1,606 cm^−1^ was assigned to the stretching vibration of C=C, and the characteristic absorbance at 1,422 cm^−1^ was attributed to the deformation vibration of CH_3_ in the ligands. The peaks located at 783 and 706 cm^−1^ were attributed to out-of-plane deviational vibrations of Ar-H, and the peaks at 1,237, 1,169, 1,059 and 868 cm^−1^ were attributed to the vibrations of Ar-C=O (deviational vibration), Ar-O-C (stretching vibration), N-H (bending vibration) and CH_2_ (rocking vibration), respectively^37^.

The atomic force microscopy (AFM) observations and morphological studies conducted using scanning electron microscopy (SEM) indicated that the block-shaped Zn-MOF microcrystals grew densely on the anodic Zn plate, forming a compact and uniform MOF film (Fig. 2b). The configuration of the prepared film was film-metal-film, which can be considered an insulator-metal-insulator device. When Ag was sputtered onto both sides of the MOF film, the geometry of the constructed device was metal-insulator-metal-insulator-metal. As shown in Supplementary Fig. 7, the Zn-MOF microcrystals could also be integrated onto indium tin oxide (ITO) glass and a silicon surface using electrochemical technology^38^, which would extend the applications of rationally designed compound 1 in electronic devices^39^,^40^.

We next investigated the characteristics and properties of the Zn-MOF film on Zn plates as an example. The UV–vis diffuse reflection spectrum confirmed that the as-prepared MOF film possessed the same band-gap value (4.23 eV) as that of the bulk MOF material (Fig. 1d).

### Morphology and thickness study

**Figure 3:**
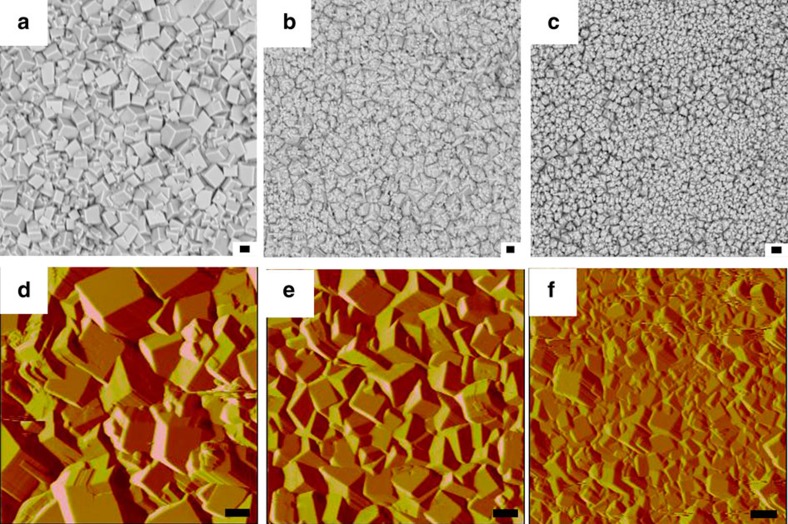
Morphology study of MOF thin films prepared at different voltages. SEM images of MOF thin films prepared at (**a**) 1 V; (**b**) 2 V; and (**c**) 3 V for 60 s and AFM deflection images of MOF thin films prepared at (**d**) 1 V; (**e**) 2 V; and (**f**) 3 V for 60 s. Scale bar, 1 μm (**a**–**f**).

**Figure 4:**
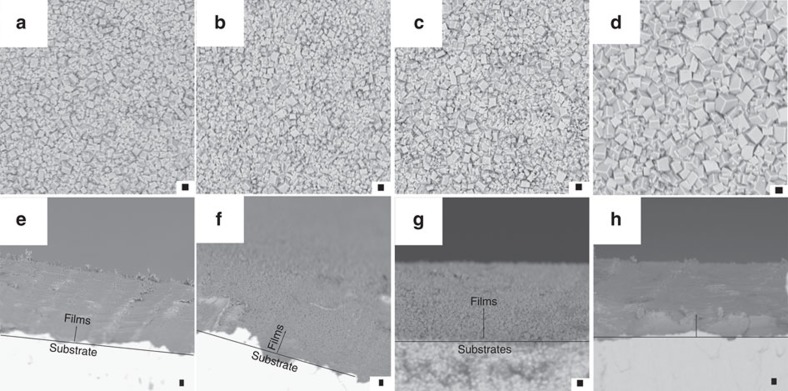
Morphology study of MOF thin films prepared using different reaction times. SEM images of MOF thin films prepared at (**a**) 420 s; (**b**) 300 s; (**c**) 180 s; and (**d**) 60 s. Images (**e**–**h**) show the cross-section views of the specimens in images (**a**–**d**) respectively. Scale bar, 1μm (**a**–**h**).

The surface morphology and film thickness of the as-prepared MOF thin films were investigated through regulation of the reaction time and voltage. As shown in Fig. 3, a series of MOF thin films, which exhibited good coalescence of microcrystals with fairly small intergranular voids, as confirmed by SEM, were obtained *via* adjusting the voltage. The particle size on the film surface decreased as the voltage increased, which may decrease the leakage current caused by large grains^41^,^42^. The roughness of the MOF films decreased from 205.71 to 150.09 nm when the reaction voltage was increased from 1 to 3 V (Fig. 3, Supplementary Fig. 8), which indicates that the morphology of the as-prepared MOF film is controllable *via* changing the reaction voltage. Moreover, the thickness of the as-prepared MOF film was tuned by changing the reaction time (Fig. 4, Supplementary Fig. 9). When the reaction time was decreased from 420 to 60 s, the thickness of the MOF film decreased from 22.0 to 5.26 μm (Supplementary Fig. 9). This result suggests that the thickness of the MOF film is controllable *via* changing the reaction time.

The charge carrier transport in gate dielectrics is strongly related to the morphology of the film. Therefore, precise control of the morphology of the film is paramount for thin-film synthesis with regards to charge carrier transport properties^41^,^42^. In addition, control of the thickness of the film is also important to decrease the leakage current. Here, we prepared films with smooth surface morphologies and preferably small intergranular voids by controlling the voltage and reaction time.

### Dielectric properties of the MOF thin film

**Figure 5:**
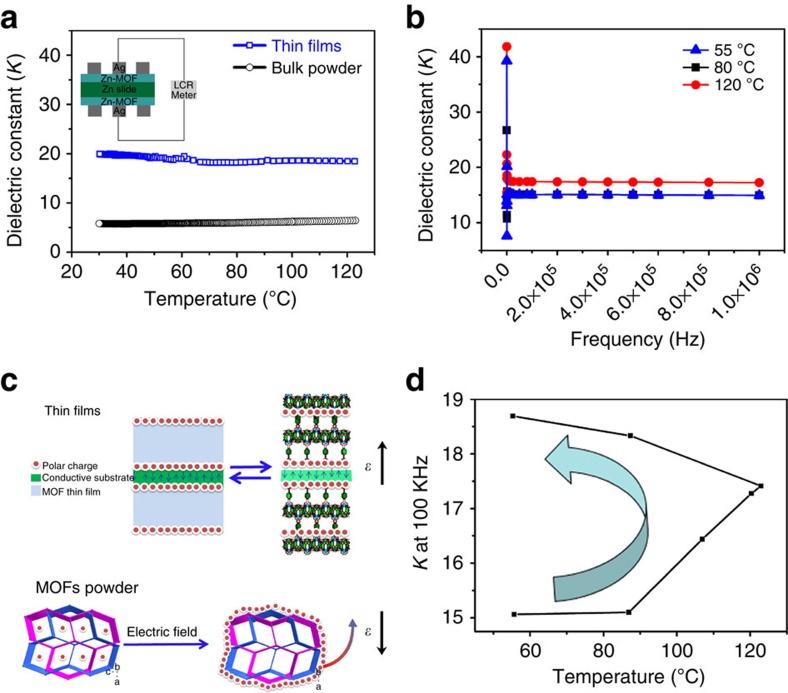
Study of dielectric behaviour of bulky 1 and MOF thin film. (**a**) κ of powdered **1** and thin films after removal of the guest molecules. (**b**) Dielectric constant κ of an MOF film during the heating process. (**c**) Schematic of the space-charge distribution in the MOF thin film and in bulk MOFs. (**d**) Representative evolution of the dielectric constant of the MOF film during a heating and cooling cycle from 55 to 125 °C. The arrow indicates the temperature sequence.

The optimized conditions consist of a voltage of 3 V and a reaction time of 60 s. The plots of the dielectric constant (*κ*) as a function of the frequency indicate that MOF thin film possesses a dielectric constant (*κ*) of 19.5 at a frequency of 1 MHz, which is much larger than those of most previously reported MOFs (except for ferroelectric MOFs)^43^,^44^,^45^. By contrast, the bulky sample of **1** only exhibits a small κ of 5.9 (Fig. 5a). The dielectric constant is a complex function that depends on the density of the material and the total polarizability of the molecules. The total polarizability is influenced by several factors, such as (a) space-charge polarization, (b) dipolar orientation and (c) displacement of electrons of atomic, ionic^46^. In the case of powder MOFs, a sluggish increase was observed as temperature was increased from room temperature to 55 °C (Supplementary Fig. 10). This phenomenon may result from the space-charge distribution that arose from the polarizability of the guest molecules in the pores of the MOF materials (Fig. 4c). However, the dielectric constant of **1** decreased with increasing temperature and remained constant at 5.8 during the cooling cycles (Supplementary Fig. 10). This decrease in the dielectric constant of **1** was most likely due to the ring distribution of the space charge weakening the total polarization of the bulky MOF (Fig. 4c)^46^. However, the measured dielectric constant of bulk **1** is still substantially larger than most of the previously reported dielectric constants for MOFs (typically less than 4.0)^47^,^48^,^49^. This result may be due to the interpenetrated structure of **1**, which would enhance the polarization of **1** and increase its dielectric constant^35^,^47^,^48^,^49^.

### Theoretical calculations

**Figure 6:**
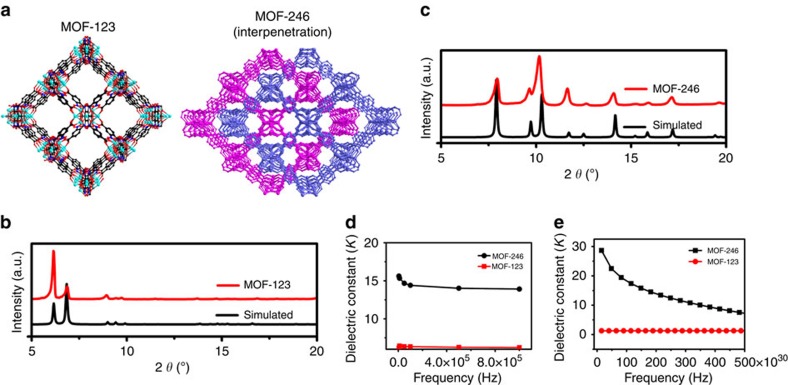
Comparative study of dielectric properties of (non-) interpenetrated MOFs. (**a**) 3D view of the non-interpenetrated structure of MOF-123 and the interpenetrated structure of MOF-246. (**b**) PXRD of MOF-123. (**c**) PXRD pattern of MOF-246. (**d**) Experimental study of the dielectric properties of MOF-123 and MOF-246 at 323.15 K. (**e**) Theoretical studies of the dielectric properties of MOF-123 and MOF-246 at 0 K.

Comprehensive experimental studies and theoretical calculations were also carried out to confirm the aforementioned conclusion concerning the increase in the dielectric constant of interpenetrated **1**. A pair of interpenetrated and non-interpenetrated MOF structures (i.e., MOF-123 (non-interpenetrated) and MOF-246 (interpenetration)) was measured to determine whether the interpenetration of the structure enhances the dielectric properties of the MOF^50^,^51^. As shown in Fig. 6, concatenated MOF-246 exhibited an experimental dielectric constant of 14.7 at 323.15 K, which is approximately 2 times larger than that of interpenetrated MOF-123 (6.3 at 323.15 K). Theoretical calculations of the dielectric constants of MOF-123 and MOF-246 were also carried out. As calculated using CASTEP MODE in Materials Studio 7.0, MOF-246 exhibited a theoretical dielectric constant of 15.5 at 0 K, which is 12 times larger than that of MOF-123 (1.3 at frequencies below 4.0 × 10^32^ Hz and at 0 K) (Fig. 6e). The results confirmed that the interpenetration of the framework can enhance the dielectric properties of MOFs, especially at low frequencies. In addition, the dielectric constants of a series of other interpenetrated MOFs were also calculated using Materials Studio for comparison to the dielectric constants of their non-interpenetrated structures. As theoretically calculated, interpenetrated MOF-5 has a dielectric constant of 423.7, which is approximately 326 times larger than that of non-interpenetrated MOF-5 (1.3) (Supplementary Fig. 11).

In the case of the fabrication of compound **1** on a conductive solid surface, the space-charge relaxation phenomenon occurred at low frequencies, as evident from the slight increase in the dissipation factor at frequencies above 10^5^ Hz (Supplementary Fig. 12). Moreover, when the temperature was increased, the dielectric constant of the MOF thin film remained ca. 19.0 at 120 °C, which is 20% larger than the dielectric constant at 55 °C (ca. 16.0). These results may be due to the increase in temperature activating the relaxation phenomenon in the MOF thin film with polar guest molecules, which will further enhance the total polarizability of the thin film and increase the dielectric constant^18^,^19^. As shown in Fig. 5d, during the heating and cooling cycles at 100 kHz, the dielectric constant of the MOF film remained at ca. 15.1 upon heating to 80 °C and then increased to 17.4 in the temperature range from 80 to 123 °C. During the cooling process from 123 to 55 °C, the dielectric constant gradually increased to ca. 18.7 (Fig. 5d) and finally stabilized at ca. 19.9. This increase in the dielectric constant may result from either the dipole moments or orientational polarization according to the Debye model^46^,^52^. However, the phenomenon observed in the thin film differs from the dielectric behaviour of bulk **1**, whose dielectric constant decreased with increasing temperature (Supplementary Fig. 10). This behaviour of the prepared MOF thin film is interesting for applications involving high-*κ* materials because the dielectric constants of the bulk MOFs and those of previously reported MOFs typically decrease during heating and cooling cycles^47^,^48^,^49^. The difference in the case of our MOF films most likely stems from the homogeneous distribution of the space charge inside the MOF particles on the two sides of the conductive substrate surface, which improves the capacitance of the entire MOF thin film and further increases its dielectric constant (Fig. 5c, equation 1). These results further indicate that the fabrication of interpenetrated MOFs on conductive substrates as thin films can enhance the dielectric constant of the MOFs, which is critical for their application as charge carriers in gate dielectrics^53^.

### Study of mechanical properties and leak current tests

**Figure 7:**
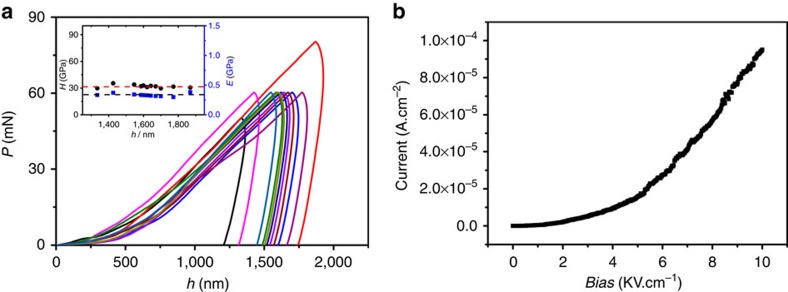
Mechanical properties and leak current measurements of the MOF films. (**a**) Representative *P* (load)-*h* (*h*=indentation depth) curves for MOF films. Inset: elastic moduli *E* of MOF films as a function of the indentation depth, wherein each error bar represents the standard deviation from 11 indents. Error bars=s.d. (**b**) Representative current-voltage curves for a MOF thin film.

Mechanical properties are important for the practical application of MOFs as semiconductors. The elastic modulus and hardness are two important parameters for the evaluation of the mechanical behaviour of films. Nanoindentation measurements were performed using a spherical diamond tip (radius=20 μm) in continuous measurement (CSM) mode (Supplementary Fig. 13) with a Poisson's ratio of 0.3 according to a previously reported protocol^54^. Representative *P*-*h* curves for the prepared thin films are shown in Fig. 7a. The load displacement of the MOF thin film is smooth, indicating that the plastic deformation that occurs underneath the spherical diamond tip during indentation is relatively homogeneous in nature. The average values of the elastic modulus (*E*) and hardness (*H*) of the MOF thin film, which were extracted from the *P*-*h* curves, are calculated for depths of 1–2 μm to avoid interference from the spherical diamond tip and substrate^54^,^55^,^56^,^57^. The average elastic modulus and hardness of this film were determined to be 32.00 (±1.71) GPa and 0.33 (±0.02) GPa, respectively, which satisfies the basic requirements for electrical devices^54^,^55^,^56^,^57^. The elastic moduli and hardness of the thin films fabricated under different conditions were also determined to be similar to these values (Supplementary Table 1). The similarity of the experimental values indicates good grain coalescence of the intergranular voids, which should improve the films' mechanical properties. The results also suggest that the as-prepared thin films can be assembled as an electronic device that can withstand large mechanical stress. As a practical gate dielectric layer, the MOF-**1** thin film exhibited a leakage current and breakdown voltage of 10^−7^ A·cm^−1^ (at 1 kV·cm^−1^) and 10 kV·cm^−1^, respectively (Fig. 7b). These results indicate that the as-prepared MOF films exhibit good insulating properties and a high breakdown strength.

## Discussion

A MOF thin film based on a 2-fold interpenetrated MOF that was constructed using a flexible ligand and metal ions was deposited onto conductive metal plates via a facile approach involving the electrochemical assembly method. The morphology and thickness of the as-prepared MOF films were controlled via tuning the reaction time and voltage. Importantly, the dielectric properties of the interpenetrated MOF thin films were systematically studied for the first time. Significantly, the interpenetrated MOF thin film exhibits a dielectric constant larger than both those of their bulk forms and those of most of the previously reported MOF materials. Furthermore, the dielectric constant of the as-prepared films was tuned through adjustment of the synthesis conditions. We propose that this remarkable increase in the dielectric constant may originate from the packing of the microcrystal polarization of the well-trapped solvent molecules and the interpenetrated framework on the conductive supports. The MOF thin films offer high mechanical flexibility and can sustain a large mechanical stress, which makes this material very promising for application in microelectronic devices.

Our research demonstrated the importance of the interpenetrating structure of the MOF thin films to achieve good dielectric and mechanical properties. This study provides a platform for the utilization of interpenetrated MOF materials in microelectronics, such as gate dielectrics and semiconductor devices.

## Methods

### Chemicals

1, 3, 5-Tris(bromomethyl)-2, 4, 6-trimethylbenzene was purchased from TCI. Ethyl-p-hydroxybenzoate and Zn(NO_3_)_2_·6H_2_O were purchased from Sinopharm Chemical Reagent Beijin Co., Ltd. They were all used without further purification. All reagents and solvents were commercially available and used as received. Ultrapure water (18.24 MΩ cm^−1^) is used directly from a Milli-Q water system. Zinc plates and ITO glasses were commercially available and used as received. AZO/silicon plates was obtained by magnetron sputtering AZO layer onto silicon surface.

### Voltage supply

Voltage supply was performed by Epsilon electrochemical workstation and Agilent E3 612A DC power supply.

### Powder X-ray diffraction measurements

PXRD test of MOF thin film was carried out on MiniFlex600 by scraping thin film from the surface of zinc plate. The simulated powder patterns were calculated using Mercury 2.0. The purity and homogeneity of the thin film were determined by comparison of the simulated and experimental X-ray powder diffraction patterns.

### Infrared spectroscopy

Attenuated total reflection infrared (ATR-IR) spectra of the samples were measured using Vertex 70 Fourier transform infrared (FTIR) spectrometer with an ATR sampling accessory. The ATR cell was made of a horizontal diamond crystal with an incidence angle of 45°.

### Scanning electron microscopy

The scanning electron microscopy (SEM) measurement was carried out on a Phenom G2 instrument. The scanning probe microscopy (SPM) measurement was carried out on a NanoscopeШa instrument.

### Energy band tests

Band gaps of samples were determined by using a Lambda 950 spectrometer working in Diffused Reflectance mode. The Kubelka-Munk equation was used to calculate absorption at the various wavelengths. Using the absorption versus energy plot, the first derivative of the absorption spectrum equation was calculated, and the inversion points could be identified.

### Dielectric permittivity measurements

The dielectric permittivity of the MOF thin film was carried out by TH2828 Precision LCR Meter. The electrodes were made by sputtering silver onto both sides of MOF film and attaching copper leads with silver paste. Calibration of standard capacitor reveals that the overall errors are less than±3% for the experimental results. The real part of the dielectric constant (κ) was calculated using the following formula (equation):


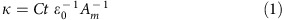


in which *C* is capacitance, *t* is the thickness of the dielectric film, *A*_*m*_ is the Silver dot area, and *ɛ*_0_ is the permittivity of vacuum.

### Theoretical calculations

Theoretical study of dielectric properties of prototypical MOFs were performed with CASTEP (Cambridge Sequential Total Energy Package)^58^,^59^,^60^ code based on the plane-wave ultrasoft pseudopotential method of Materials Studio 7.0 (ref. 61). The exchange and correlation functional was treated by generalized gradient approximation(GGA)—Perdew-Burke-Ernzerhof. The cutoff energy of 351.0 eV and *k*-points of 1 × 1 × 1 for all hypothetical structures were applied to calculate the total energy.

### Nanoindentation and leakage current measurements

Nanoindentation measurement was carried out on a MTS XP instrument. The leakage current and breakdown voltage were measured by KEITHLETY-2400 instrument.

### Thermogravimetric analyses

Thermogravimetric analyses (TGA) were performed under a nitrogen atmosphere with a heating rate of 10 °C/min using NETZSCH STA 449F3 thermogravimetric analyzer. H NMR spectra were recorded at ambient temperature on a BRUKER AVANCE III spectrometer; the chemical shifts were referenced to TMS in the solvent signal in *d*_6_-DMSO.

### Synthesis of {Zn(TBTC)[H_2_N(CH_3_)_2_]}·2DMF·EtOH (1)

The 1, 3, 5-tris[4-(carboxyphenyl)oxamethyl]-2, 4, 6-trimethylbenzene (H_3_TBTC) ligand was synthesized as following^18^,^62^: Under a nitrogen atmosphere, a dimethylformamide (DMF) (80 ml) solution of 1, 3, 5-Tris(bromomethyl)-2, 4, 6-trimethylbenzene (2.55 g, 6.4 mmol), ethyl-p-hydroxybenzoate (4.00 g, 24.1 mmol), and anhydrous potassium carbonate (K_2_CO_3_) (10.95 g, 79.2 mmol) was heated under vigorous stirring at 90 °C for 96 h. The obtained mixture was cooled to room temperature, and the solvent was removed under reduce pressure. Ultrapure water (H_2_O) (100 ml) was added to mixture, and extraction with dichloromethane (CH_2_Cl_2_) three times. Then, the intermediate ester was obtained after removing the CH_2_Cl_2_. Subsequently, under a nitrogen atmosphere, the intermediate ester (3.00 g, 6.6 mmol) was dissolved in a refluxing mixture of 80 ml of ethanol (EtOH) and 60 ml tetrahydrofuran (THF). To this solution was added 50 ml of 20% KOH solution. After 2 h, the mixture was cooled to room temperature and concentrated to dryness. The obtained residue was dissolved in 30% aqueous EtOH (40 ml) and acidified with glacial acetic acid to give the partially protonated product which precipitated from solution. This precipitated was collected and dissolved in THF (30 ml) and trimethylsilyl chloride (1.73 g) was added to the solution. The mixture was stirred at room temperature for half an hour and precipitated into water (300 ml). The precipitate of fully protonated product was collected by suction filtration and dried to get H_3_TBTC ligand. Zn(NO_3_)_2_·6H_2_O (2.00 mmol, 0.590 g) and H_3_TBTC (1.00 mmol, 0.570 g) were dissolved in a mixture consisting of 10 ml of 4:1:1 (volume ratio) DMF/Ethanol/H_2_O with stirring and heating for 20 min. The solution was then transferred to a Teflon-lined stainless steel vessel (23 ml). After heating under an autogenous pressure at 120 °C for 72 h, the Teflon-lined stainless-steel vessel was slowly cooled to room temperature at a constant rate of 0.04 °C·min^−1^. Colourless crystals of **1** were obtained and dried in air at ambient temperature.

### Single-crystal X-ray crystallography

Crystal data for {[H_2_N(CH_3_)_2_][Zn(TBTC)]}·2DMF·EtOH (**1**) (Supplementary Table 2, Supplementary Table 3), orthorhombic, *P*_*bcn*_, *Mr*=676.99 Å, *a*=29.2649(4) Å, *b*=7.89750(10) Å, *c*=42.5654(7) Å, *α*=90.00°, *β*=90.00°, *γ*=90.00°, *V*=9837.7(2) Å^3^, *D*_calcd_=0.914 g·cm^−3^. *μ*=1.006 mm^−1^, *F*(000)=2,816, *GOF*=1.084. A total of 36,658 reflections were collected, 8,596 of which were unique (*R*_*int*_=0.0697). *R*1/*ωR*_2_= 0.0809/0.2105 for 415 parameters and 8,596 reflections (*I*>2*σ*(*I*)). Measurement of crystal was conducted on SuperNova diffractometer which equipped with a copper microfous X-ray source (*λ*=1.5406 Å) at 100(3) K. The structure was solved by direct methods and refined on *F*^2^ by full-matrix least-squares using the SHELXL-97 program package^63^. The positions of H atoms were generated geometrically. Non-hydrogen atoms were refined with anisotropic displacement parameters. Given that refinement of the metal-organic coordination framework is essentially uninfluenced by the presence of the disordered solvent and lattice solvent molecules were badly disordered, the SQUEFZE routine in the PLATON software was applied to subtract the diffraction contribution from the solvent molecules^35^. The number of free DMF molecules per unit cell was estimated from the TGA or ^1^H NMR.

### Electrochemical assembly of MOF thin films

H_3_TBTC (1.00 mmol, 0.570 g) and ammonium fluoride (NH_4_F, 2.7 mmol, 0.100 g) were dissolved in a mixture solution of 100 ml of 4:1:1 (volume ratio) DMF/EtOH/H_2_O with stirring for 10 min under heating. The solution was then bubbled with N_2_ (approximately three bubbles per second) for 1 h to remove the oxygen from the solution. Two Zn-plate electrodes were then immersed into the solution. The MOF film was grown on the Zn plate serving as the anode by applying a voltage while maintaining continuous N_2_ bubbling through the solution. The as-prepared MOF film was washed thoroughly with DMF to remove excess ligand and NH_4_F, respectively. MOF films with different morphologies were prepared using different voltages and reaction times. With respect to the deposition of compound **1** on ITO glass and AZO/silicon surface (AZO, aluminium-doped zinc oxide, the width and length were approximately 10 × 30 mm), Zn (NO_3_)_2_·6H_2_O (2.00 mmol, 0.590 g) and H_3_TBTC (1.00 mmol, 0.570 g) were dissolved in 4:1:1 (volume ratio) DMF/EtOH/H_2_O, respectively. The Zn^2+^ solution was then slowly poured into the TBTC^3−^ solution under heating, and colloidal compound **1** nanocrystals were formed in solution. The colloidal nanocrystals were dispersed into a methylbenzene solution or methanol solution. The films were electrochemically obtained by inserting two ITO glass electrodes or AZO/silicon plates into the colloidal nanocrystal solutions under an applied voltage.

### Data availability

Crystallographic data (excluding structure factors) for the structure reported in this paper have been deposited at the Cambridge Crystallographic Data Center, under the deposition number 1043020. Copies of the data can be obtained free of charge via www.ccdc.cam.ac.uk/data_request/cif. The data that support the finding of this study are available from the corresponding author upon request.

## Additional information

**How to cite this article:** Li, W.-J. *et al*. Integration of metal-organic frameworks into an electrochemical dielectric thin film for electronic applications. *Nat. Commun.* 7:11830 doi: 10.1038/ncomms11830 (2016).

## Supplementary Material

Supplementary Information Supplementary Figures 1-13 and Supplementary Tables 1-3

Supplementary Data 1 Crystallographic Information File for **1**.
