# Supplementary material for: Integration of metal-organic frameworks into an electrochemical dielectric thin film for electronic applications
Source: Nat Commun. 2016 Jun 10;7:11830. doi: 10.1038/ncomms11830 (PMC4906389; doi:10.1038/ncomms11830)
Supplement: Supplementary Information — Supplementary Figures 1-13 and Supplementary Tables 1-3 [file ncomms11830-s1.pdf]

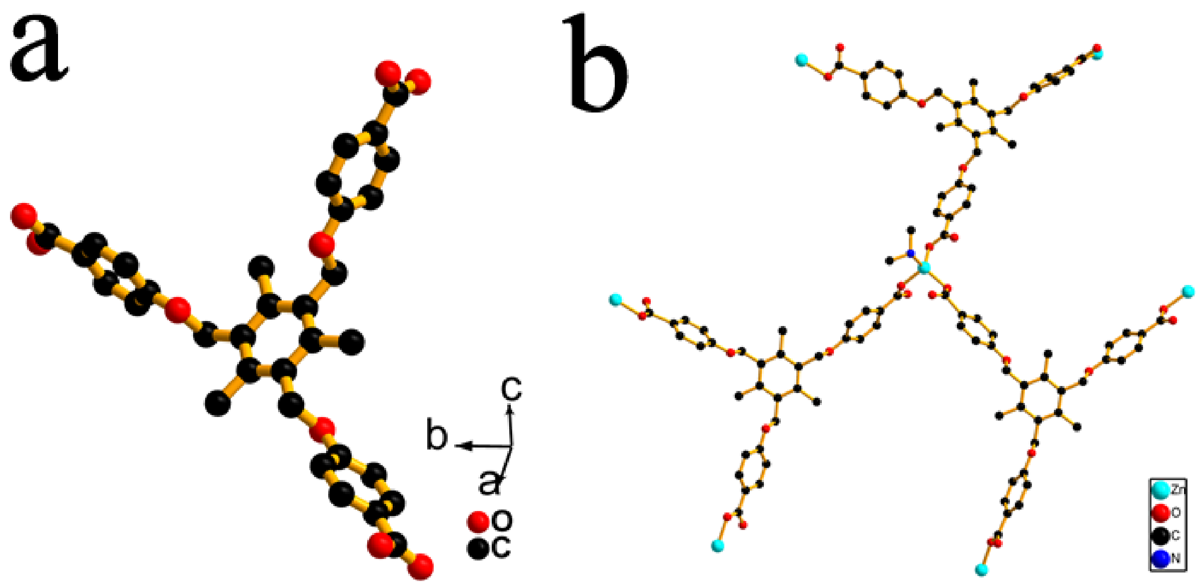

Supplementary Figure 1 | The constitutional units and the coordination envrionment of compound 1. (a) Ligand of the compound **1**. (b) Coordination environment of compound **1**.

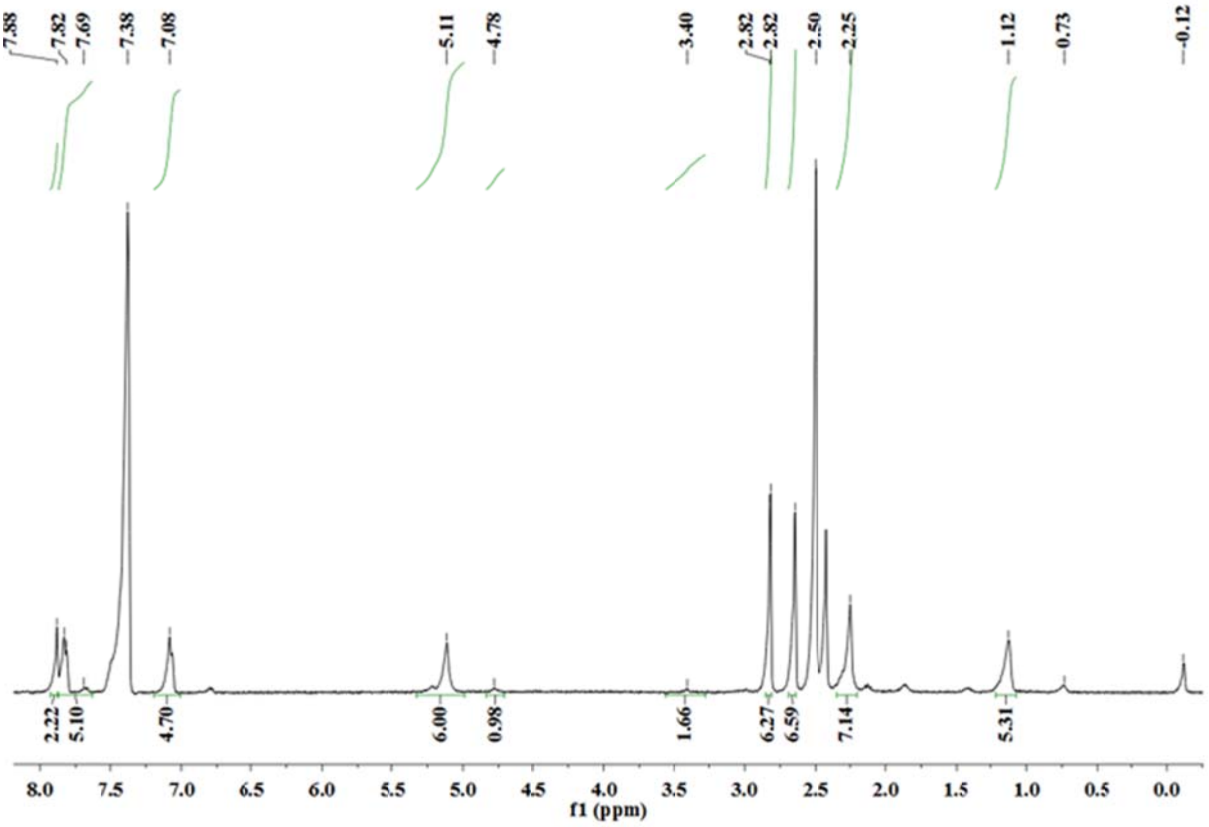

Supplementary Figure 2 | The ^{1}H NMR of solvents in the pores of compound 1. A H NMR study was performed to determine the number of guest molecules per $[H_2N(CH)_3^+]Zn(TBTC)$ unit for compound **1**.～5 mg of compound **1** was completely dissolved in 20 μL of d_6-DMSO. H-NMR (400 MHz) spectra of dissolved samples were collected at room temperature. The integration for one set of the H_3TBTC was set of 6 for the $[H_2N(CH)_3^+]Zn(TBTC)$. We then integrated the methyl, methylene, amide peak for DMF and methyl, methylene, hydroxyl peak for ethanol. The data are consistent with TGA and are summarized below:

DMF: CH_3 δ2.68 (triplet, 6.59 H)

CH_3δ2.82 (triplet, 6.27 H)

CH δ7.88 (singlet, 2.22 H)

Ethanol: CH_3 δ1.12 (triplet, 5.31 H)

CH_2 δ3.40 (quartet, 1.66 H)

OH δ4.78 (singlet, 0.98 H)

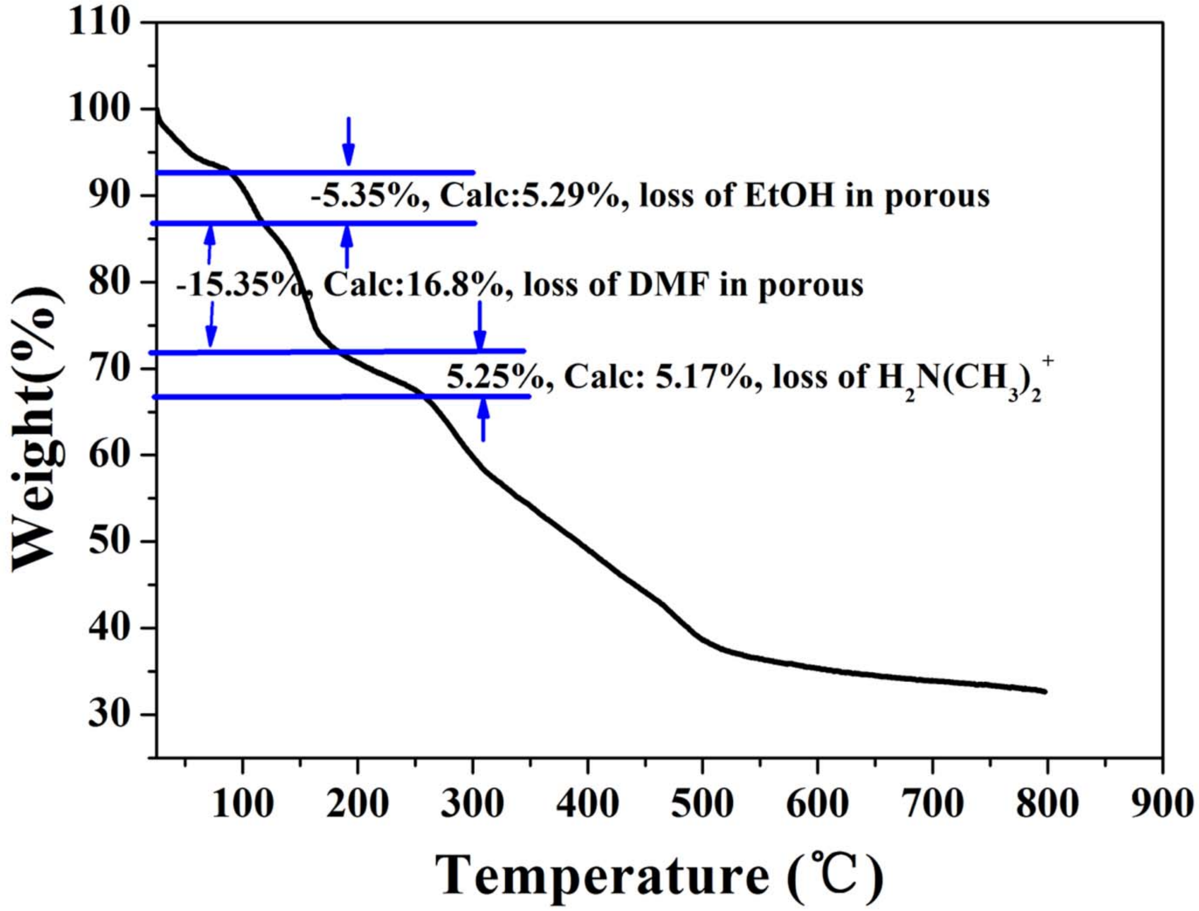

Supplementary Figure 3 | TG curve of compound 1. The first weight losses of 5.35% in the range of 30 ℃~120 ℃ is related to the loss of ethanol molecules (calculated at 5.29%). The second weight loss of 15.35% is related to the loss of DMF molecules in porous (calculated at 16.8%). The third weight loss of 5.25% is related to the loss of $H_2N(CH)_3^+$ units (calculated at 5.17%).

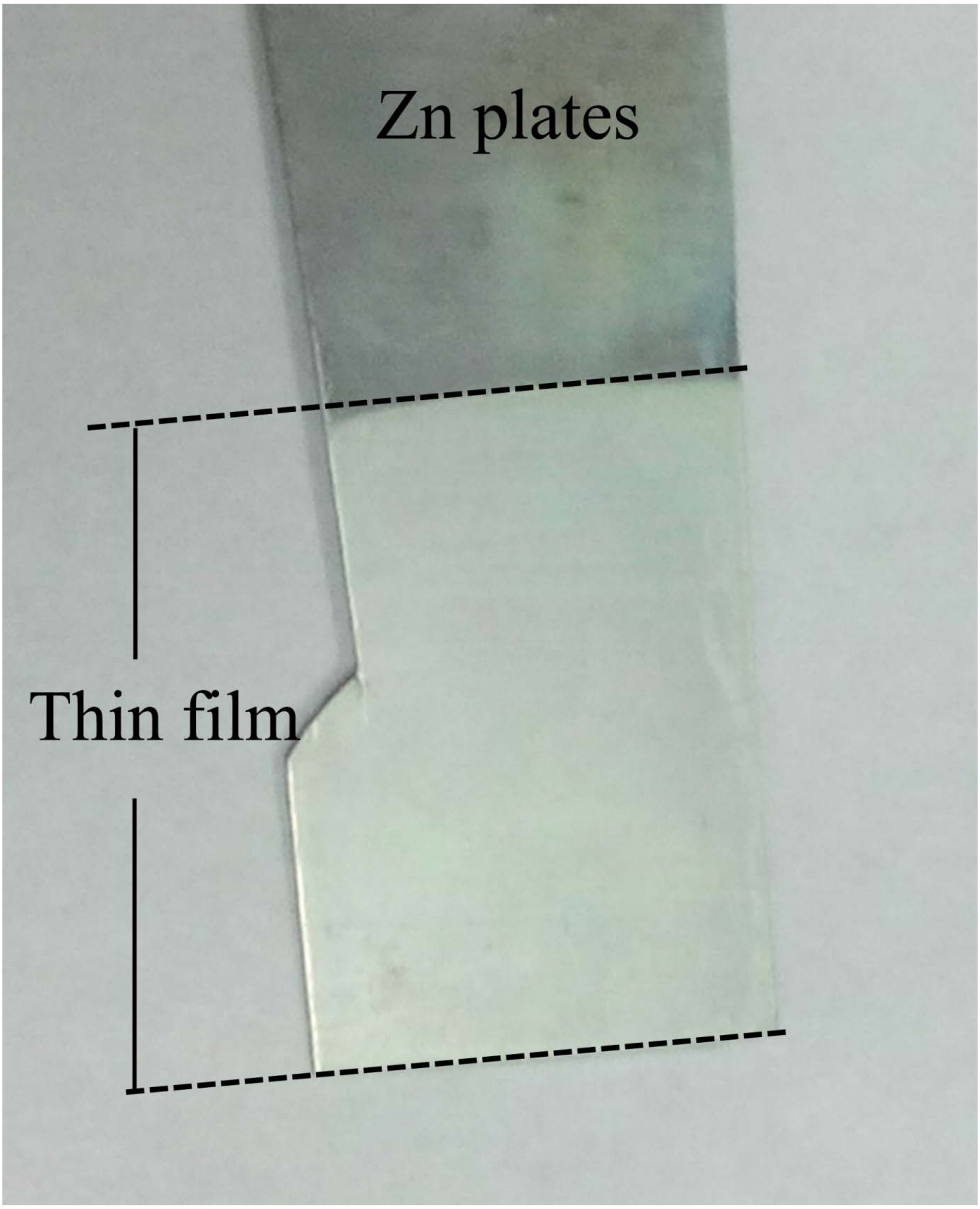

Supplementary Figure 4 | Optical images of the prepared MOF thin film. The size of the plates is approximately 10 mm × 30 mm, width × length. The flatness can be clearly seen from the displayed optical images.

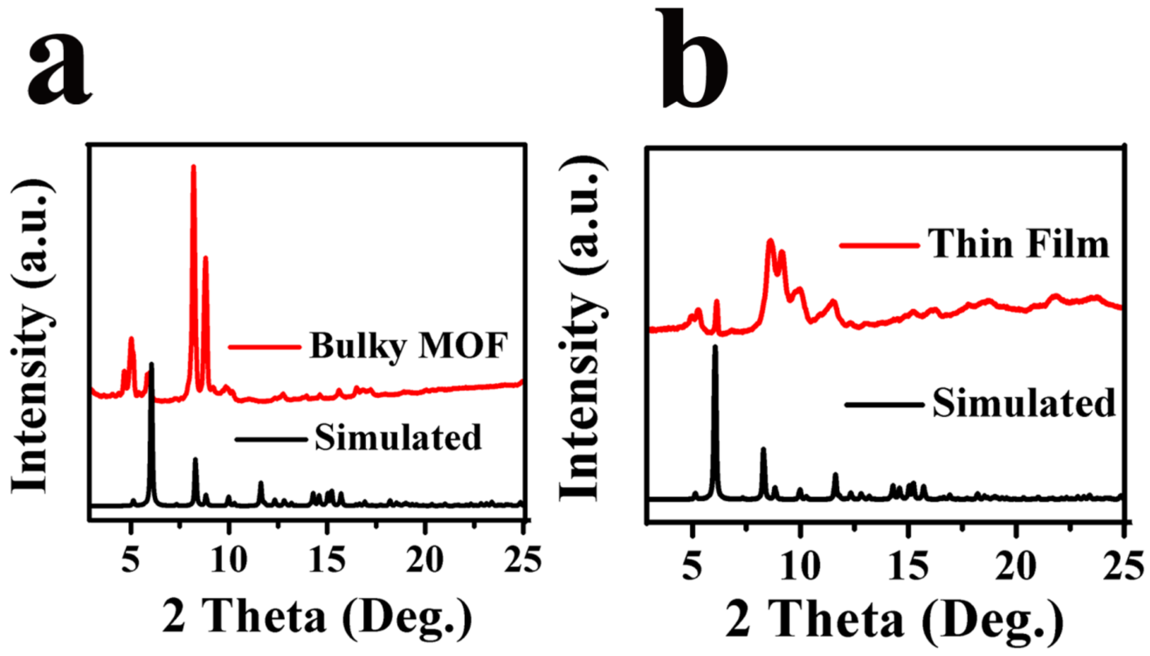

Supplementary Figure 5 | PXRD studies of bulky MOFs and thin film. (a) PXRD of bulky powder of **1**. (b) PXRD of thin film. The high noise and broad peaks of PXRD of the thin film may be attributed to small amounts of MOFs microcrystals on the solid surface.

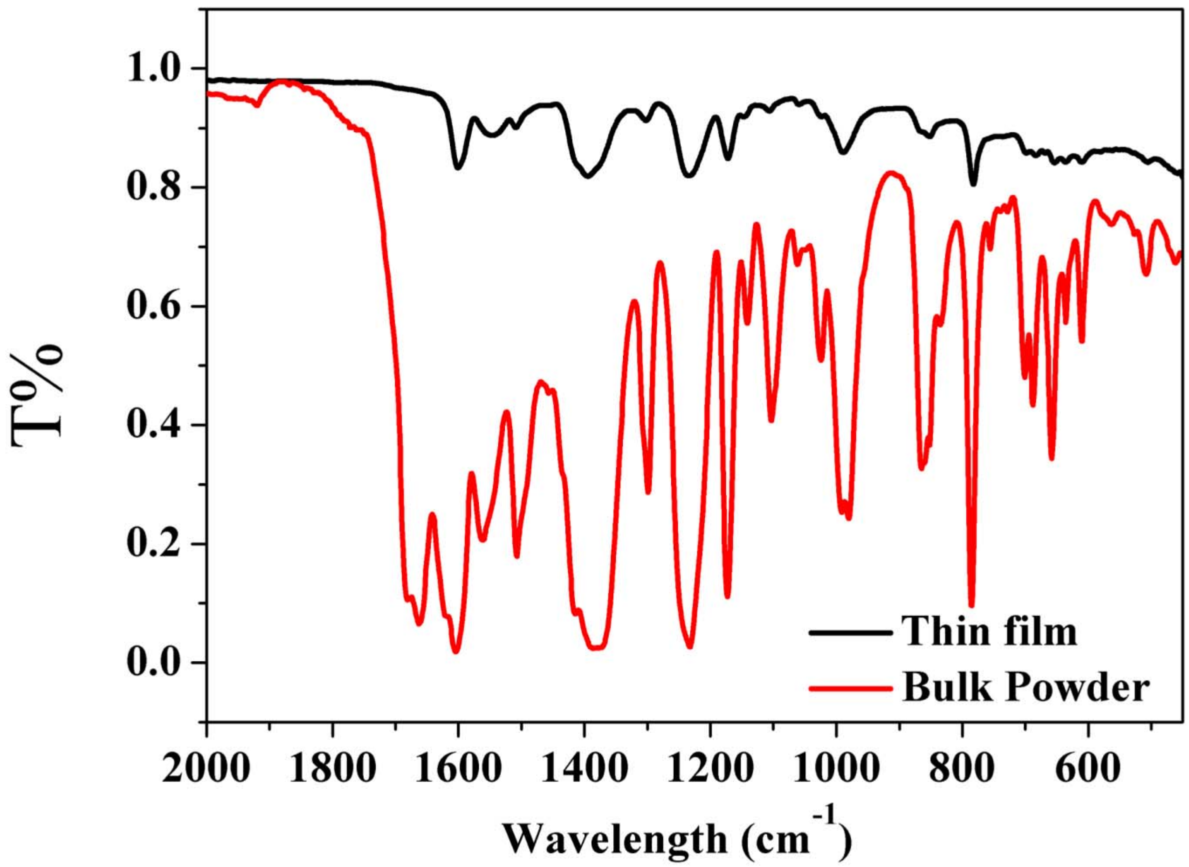

Supplementary Figure 6 | IR characteristic of the prepared thin films. FT-IR spectra of bulky compound **1** and ATR-IR of MOF thin film.

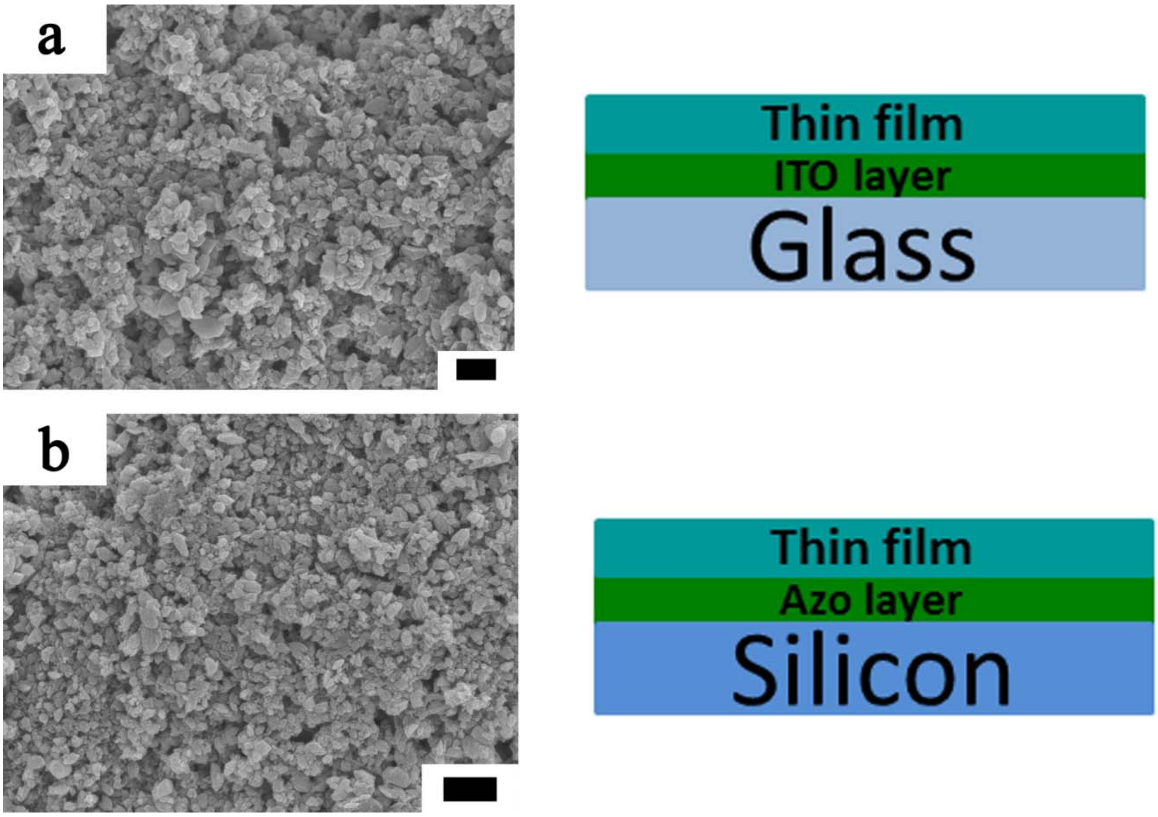

Supplementary Figure 7 | SEM images of 1 deposited onto other conductive surface. (a) SEM images and schematic of compound **1** deposited on ITO surface. (b) SEM images and schematic of compound **1** deposited on AZO/silicon surface. Scale bar, 1 μm.

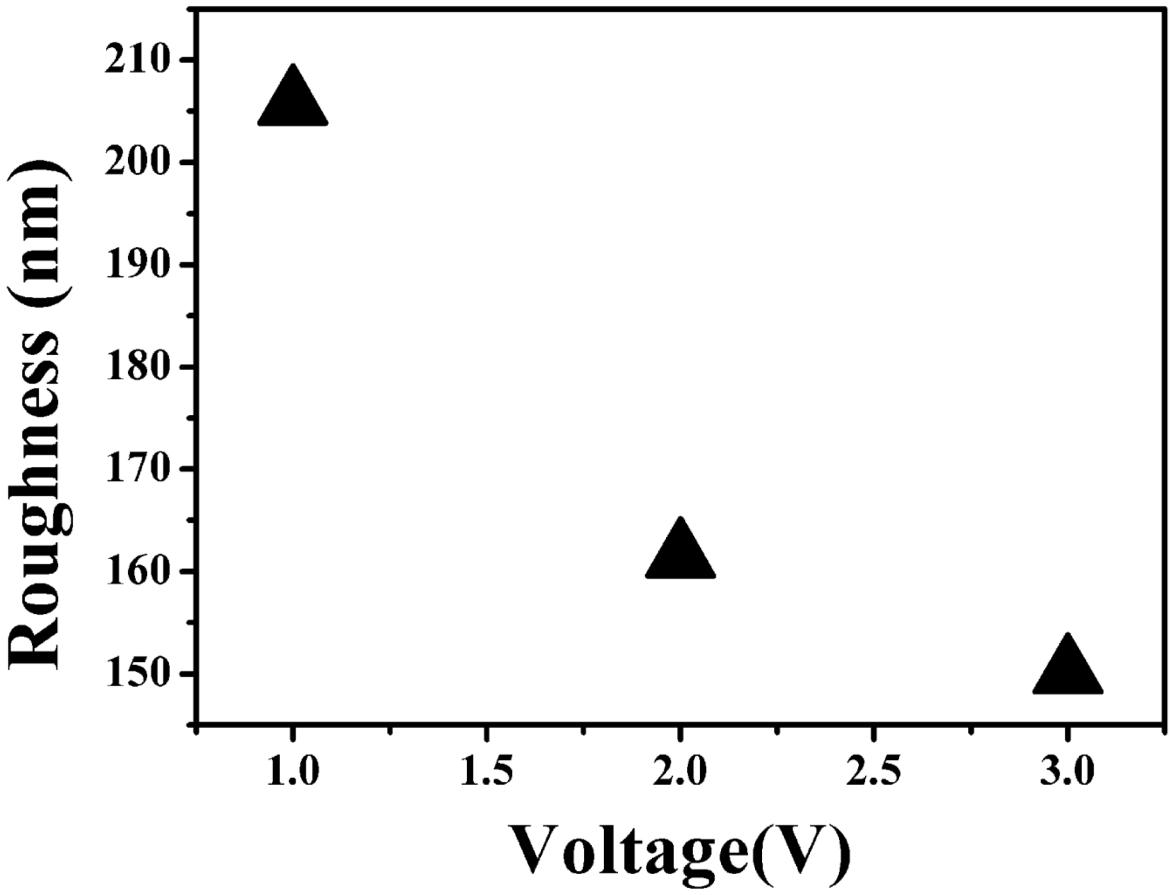

Supplementary Figure 8 |Roughness of MOF thin films. Plotted as roughness of MOF thin film prepared at 1V (205.71 nm), 2 V (161.43 nm) and 3 V (150.09 nm) for 60 s.

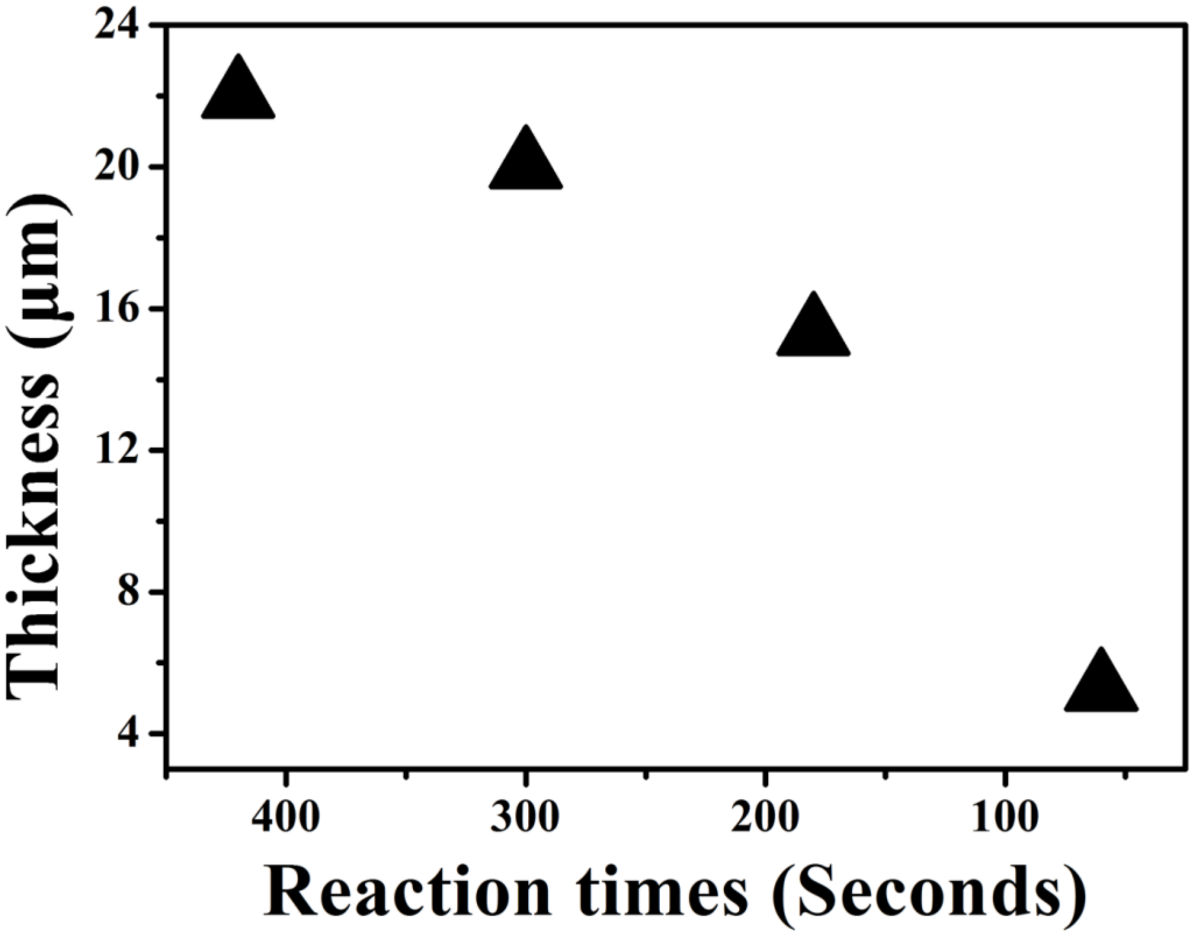

Supplementary Figure 9 | Thickness of MOF thin films. Plotted as thickness of MOF thin film prepared at 1V for different times: 420s (22.0 µm), 300 (20.0 µm), 180s (15.3 µm), 60s (5.26 µm).

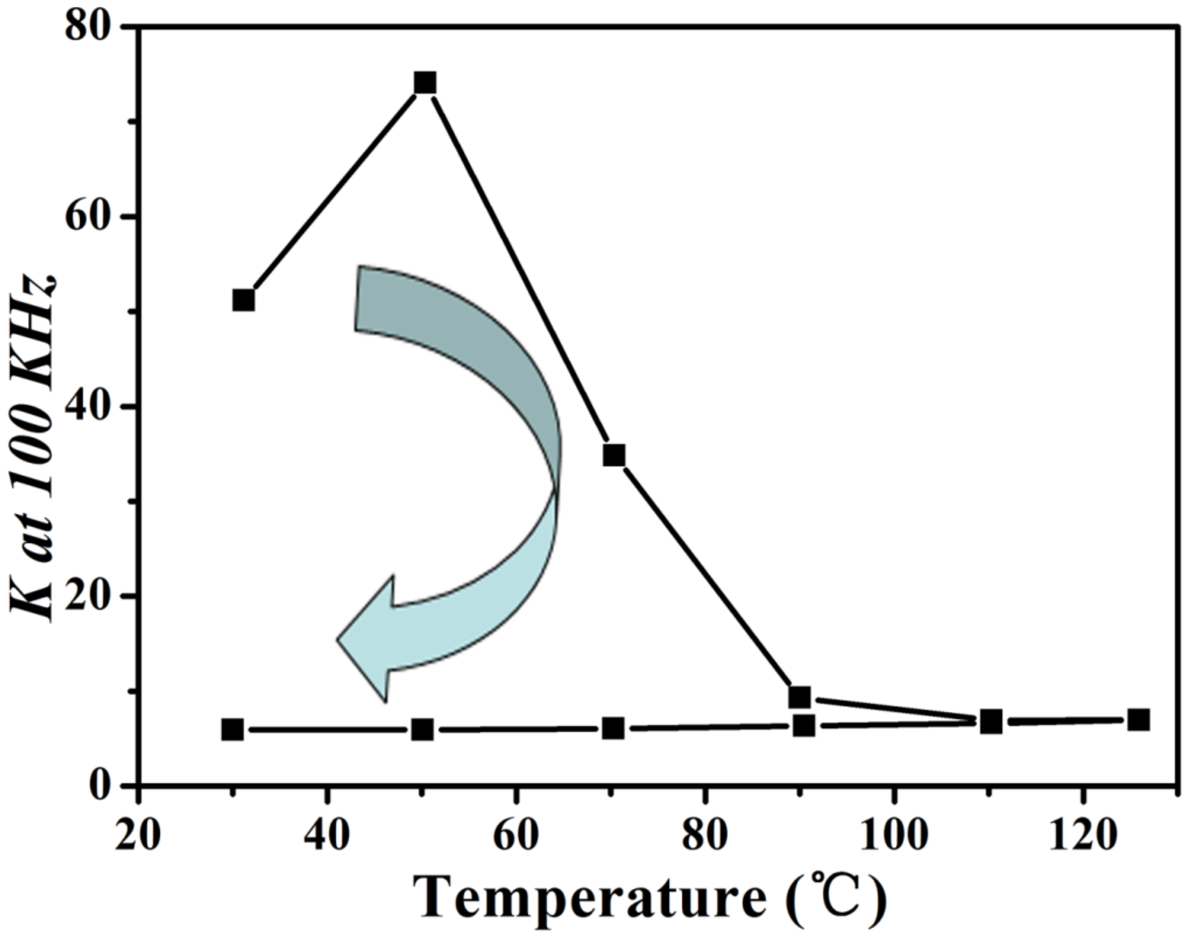

Supplementary Figure 10 | Dielectric behavior of 1 in heating and cooling cycle. Monitoring dielectric constant of bulky MOF in heating and cooling cycle at 100 Hz. Arrow indicates the temperature sequence.

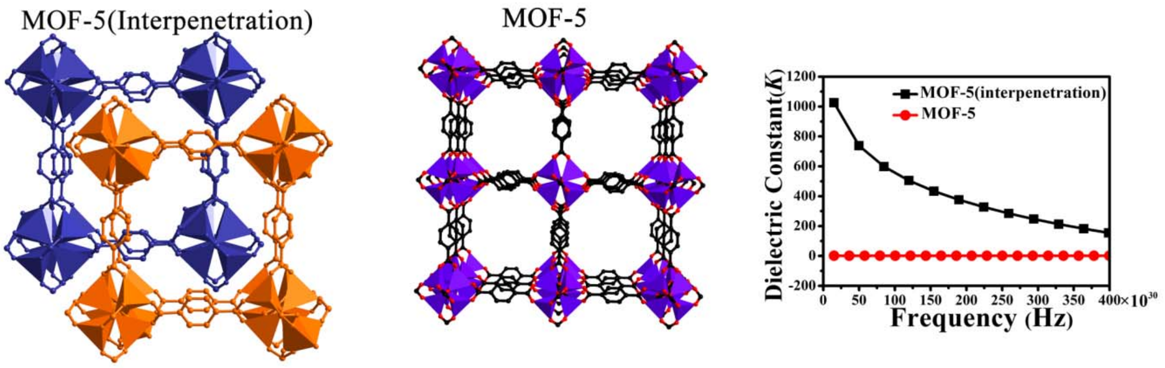

Supplementary Figure 11 | Theoretic study of dielectric properties of (non-) interpenetration MOF-5. The dielectric constant of interpenetrated MOF-5 is caculated as 423.7 (average value), while the dielectric constant of non-interpenetrated MOF-5 is caculated as 1.3 (average value).

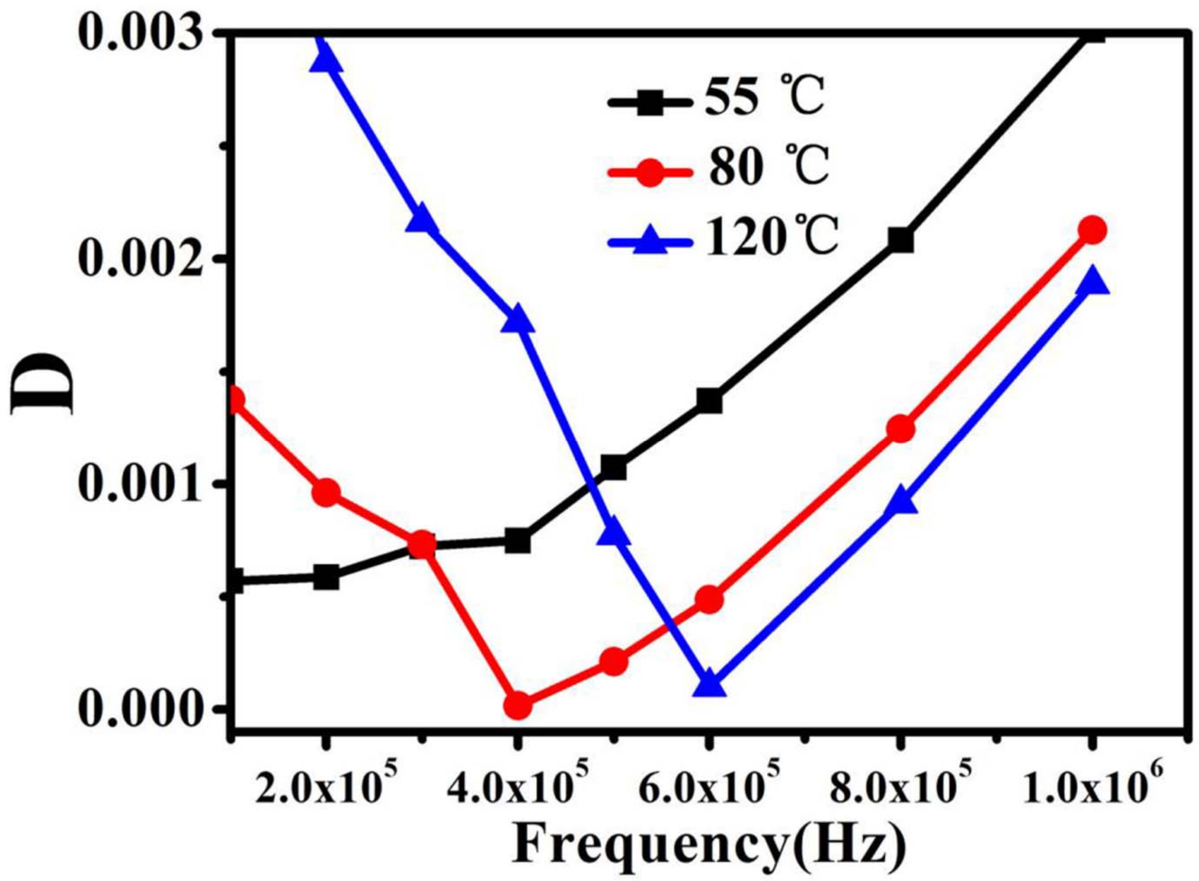

Supplementary Figure 12 | Dielectric loss of the prepared thin films in high frequency. D of CP film above the frequency of 2.0×10^5 Hz.

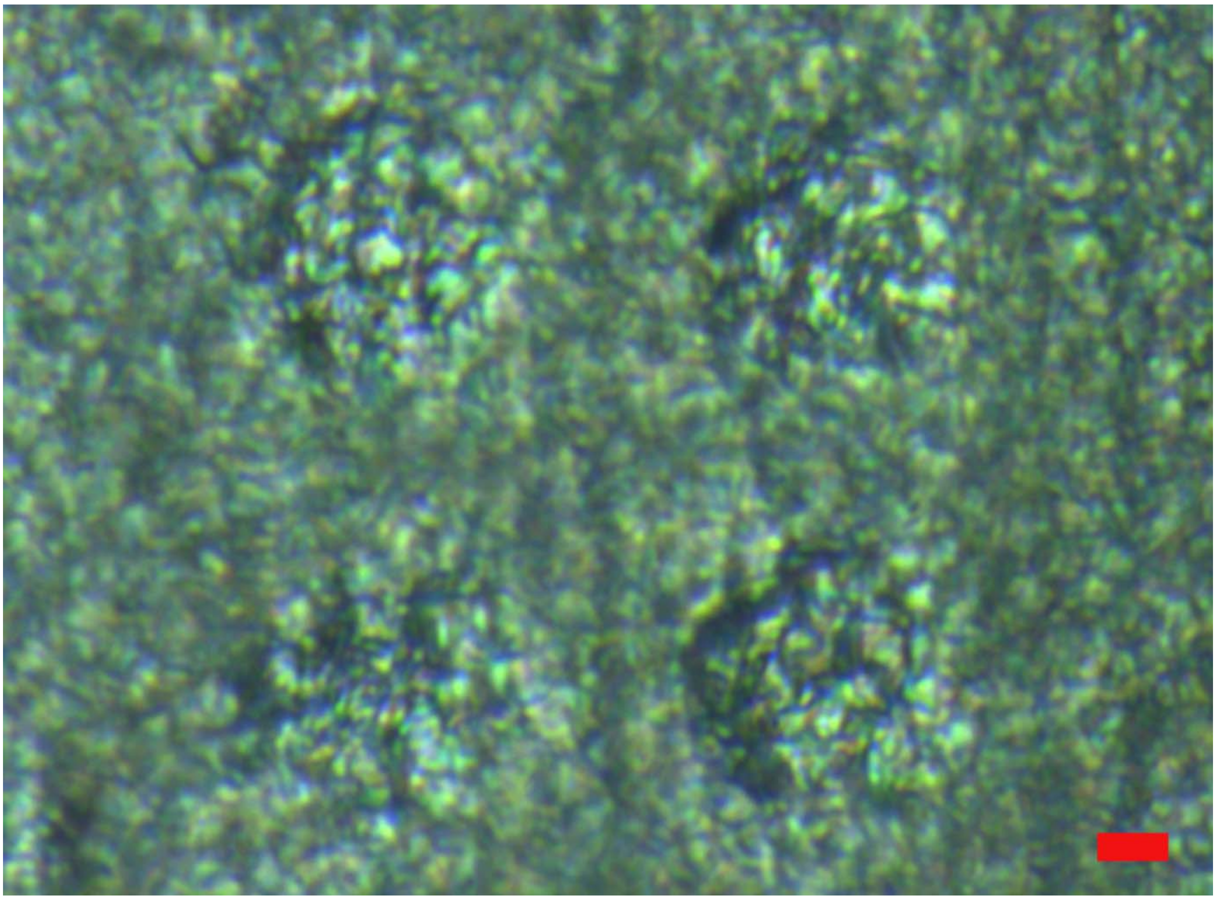

Supplementary Figure 13 | The image of nano indentation test. The trace of spherical diamond tip can be obviously seen from the optical image. Scale bar, 3 µm.

Supplementary Table 1 | Elastic moduli and hardness for thin films. Indentation depth was determined according to film thickness and roughness. Generally, the penetration depth was determined below 10% of film thickness and larger than 20 times of roughness (Ra). Error bars=s.d.

Reaction condition	E(GPa)	H(GPa)
Prepared at 5min	32.00(±1.71)	0.33(±0.02)
Prepared at 7min	32.28(±3.92)	0.44(±0.04)
Prepared at 9min	33.38(±1.88)	0.38(±0.02)

Supplementary Table 2 | Crystal data and structure refinement for crystal 1.

Identification code	$\{Zn^{2+}[H_2N(CH_3)^2]^+(TBTC)^{3-}\}\cdot xDMF\cdot xH_2O\cdot x$EtOH
Empirical formula	$C_{35}H_{33}NO_9Zn$
Formula weight	676.99
Temperature	100.0(3) K
Wavelength	1.54184 Å
Crystal system	Orthorhombic
Space group	*P*bcn
Unit cell dimensions	*a* = 29.2649(4) Å α = 90°
	b = 7.89750(10) Å β = 90°
	c = 42.5654(7) Å γ = 90°
Volume/Z	9837.7(2) $Å^3$/8
Density (Calculated)	0.914 $Mg\cdot m^{-3}$
Absorption coefficient	1.006 mm^{-1}
F(000)	2816
Crystal size	0.2 x 0.1 x 0.1 mm
Theta range for data collection	3.02 to 66.51 deg.
Limiting indices	-34<=h<=31, -9<=k<=8, -50<=l<=50
Reflections collected	36658
Independent reflections	8596 (*R*(int) = 0.0371)
Completeness to theta	66.51 degree 99.00%
Absorption correction	Semi-empirical from equivalents
Max. and min. transmission	1.00000 and 0.75059
Refinement method	Full-matrix least-squares on F^2
Data / restraints / parameters	8596 / 0 / 415
Goodness-of-fit on F^2	1.084
Final R indices [I>2sigma(I)]	*R*1 = 0.0697, w*R*2 = 0.2013
R indices (all data)	*R*1 = 0.0809, w*R*2 = 0.2105
Largest diff. peak and hole	1.582 and -0.477 eA^{-3}

Supplementary Table 3| Bond lengths [Å] and angles [°] for crystal 1.

Zn(1)-O(1)	1.958(2)
Zn(1)-O(7)#1	1.968(2)
Zn(1)-O(4)#2	2.009(4)
Zn(1)-N(1)	2.069(3)
Zn(1)-C(12)#2	2.496(8)
O(9)-C(27)	1.367(4)
O(9)-C(30)	1.432(4)
O(3)-C(5)	1.357(4)
O(3)-C(8)	1.442(3)
O(1)-C(1)	1.280(4)
O(6)-C(16)	1.375(4)
O(6)-C(19)	1.439(4)
C(9)-C(10)	1.407(4)
C(9)-C(32)	1.409(4)
C(9)-C(8)	1.512(4)
O(8)-C(23)	1.259(5)
C(31)-C(21)	1.411(4)
C(31)-C(32)	1.406(4)
C(31)-C(30)	1.497(4)
O(2)-C(1)	1.233(4)
C(27)-C(26)	1.388(5)
C(27)-C(28)	1.399(5)
C(21)-C(20)	1.400(4)
C(21)-C(22)	1.510(5)
C(32)-C(33)	1.512(4)
C(20)-C(10)	1.408(4)
C(20)-C(19)	1.493(5)
C(7)-C(6)	1.376(5)
C(7)-C(2)	1.399(5)
C(1)-C(2)	1.492(5)
C(5)-C(4)	1.395(4)
C(5)-C(6)	1.398(4)
N(1)-C(35)	1.448(5)
N(1)-C(34)	1.492(5)
C(2)-C(3)	1.399(5)
C(10)-C(11)	1.502(4)
C(3)-C(4)	1.374(5)
C(28)-C(29)	1.381(5)
C(24)-C(25)	1.378(5)
C(24)-C(29)	1.393(5)
C(24)-C(23)	1.499(5)
C(25)-C(26)	1.390(5)
C(17)-C(16)	1.345(7)
C(17)-C(18)	1.485(8)
O(5)-C(12)	1.188(7)
C(16)-C(15)	1.363(7)
C(18)-C(13)	1.370(8)

C(13)-C(14)	1.263(8)
C(13)-C(12)	1.728(9)
C(15)-C(14)	1.379(7)
C(23)-O(7)	1.250(5)
O(7)-Zn(1)#3	1.968(2)
C(12)-O(4)	1.148(6)
C(12)-Zn(1)#4	2.496(8)
O(4)-Zn(1)#4	2.009(4)
O(1)-Zn(1)-O(7)#1	108.65(11)
O(1)-Zn(1)-O(4)#2	111.61(14)
O(7)#1-Zn(1)-O(4)#2	100.99(12)
O(1)-Zn(1)-N(1)	108.90(11)
O(7)#1-Zn(1)-N(1)	100.87(12)
O(4)#2-Zn(1)-N(1)	124.00(15)
O(1)-Zn(1)-C(12)#2	136.09(15)
O(7)#1-Zn(1)-C(12)#2	97.87(18)
O(4)#2-Zn(1)-C(12)#2	26.83(16)
N(1)-Zn(1)-C(12)#2	99.30(17)
C(27)-O(9)-C(30)	117.3(2)
C(5)-O(3)-C(8)	116.4(2)
C(1)-O(1)-Zn(1)	109.9(2)
C(16)-O(6)-C(19)	115.6(3)
C(10)-C(9)-C(32)	120.7(3)
C(10)-C(9)-C(8)	118.2(3)
C(32)-C(9)-C(8)	121.1(3)
C(21)-C(31)-C(32)	120.5(3)
C(21)-C(31)-C(30)	118.6(3)
C(32)-C(31)-C(30)	120.9(3)
O(9)-C(27)-C(26)	116.5(3)
O(9)-C(27)-C(28)	123.8(3)
C(26)-C(27)-C(28)	119.6(3)
O(9)-C(30)-C(31)	109.1(2)
C(20)-C(21)-C(31)	119.7(3)
C(20)-C(21)-C(22)	120.8(3)
C(31)-C(21)-C(22)	119.4(3)
C(9)-C(32)-C(31)	119.2(3)
C(9)-C(32)-C(33)	120.0(3)
C(31)-C(32)-C(33)	120.7(3)
C(21)-C(20)-C(10)	120.4(3)
C(21)-C(20)-C(19)	121.1(3)
C(10)-C(20)-C(19)	118.4(3)
C(6)-C(7)-C(2)	121.4(3)
O(2)-C(1)-O(1)	123.0(3)
O(2)-C(1)-C(2)	120.8(3)
O(1)-C(1)-C(2)	116.1(3)
O(3)-C(5)-C(4)	116.0(3)
O(3)-C(5)-C(6)	124.9(3)
C(4)-C(5)-C(6)	119.1(3)
C(7)-C(6)-C(5)	120.0(3)
C(35)-N(1)-C(34)	110.3(3)

C(35)-N(1)-Zn(1)	111.4(2)
C(34)-N(1)-Zn(1)	115.7(2)
O(3)-C(8)-C(9)	107.6(2)
C(3)-C(2)-C(7)	117.8(3)
C(3)-C(2)-C(1)	122.3(3)
C(7)-C(2)-C(1)	119.9(3)
C(9)-C(10)-C(20)	119.4(3)
C(9)-C(10)-C(11)	119.8(3)
C(20)-C(10)-C(11)	120.7(3)
C(4)-C(3)-C(2)	121.2(3)
C(29)-C(28)-C(27)	119.1(3)
C(25)-C(24)-C(29)	118.1(3)
C(25)-C(24)-C(23)	121.8(3)
C(29)-C(24)-C(23)	120.0(3)
C(28)-C(29)-C(24)	121.9(3)
C(26)-C(25)-C(24)	121.2(3)
C(3)-C(4)-C(5)	120.4(3)
O(6)-C(19)-C(20)	107.8(3)
C(25)-C(26)-C(27)	120.0(3)
C(16)-C(17)-C(18)	115.6(5)
C(17)-C(16)-C(15)	120.0(4)
C(17)-C(16)-O(6)	114.6(4)
C(15)-C(16)-O(6)	125.4(4)
C(13)-C(18)-C(17)	119.5(5)
C(14)-C(13)-C(18)	122.5(6)
C(14)-C(13)-C(12)	120.9(5)
C(18)-C(13)-C(12)	116.6(5)
C(16)-C(15)-C(14)	123.2(6)
C(13)-C(14)-C(15)	119.0(7)
O(7)-C(23)-O(8)	125.9(3)
O(7)-C(23)-C(24)	116.2(3)
O(8)-C(23)-C(24)	117.8(3)
C(23)-O(7)-Zn(1)#3	133.7(2)
O(4)-C(12)-O(5)	139.5(8)
O(4)-C(12)-C(13)	109.6(5)
O(5)-C(12)-C(13)	108.9(6)
O(4)-C(12)-Zn(1)#4	52.2(4)
O(5)-C(12)-Zn(1)#4	88.1(5)
C(13)-C(12)-Zn(1)#4	161.2(4)
C(12)-O(4)-Zn(1)#4	101.0(5)

Symmetry transformations used to generate equivalent atoms:
#1 x,-y+3,z+1/2 #2 x+1/2,y+1/2,-z-1/2
#3 x,-y+3,z-1/2 #4 x-1/2,y-1/2,-z-1/2
